# Comparative Analyses of Antibiotic Resistance Genes in Jejunum Microbiota of Pigs in Different Areas

**DOI:** 10.3389/fcimb.2022.887428

**Published:** 2022-05-26

**Authors:** Yongxiang Li, Yuting Yang, Li Ma, Jianping Liu, Qingcong An, Chunyong Zhang, Gefen Yin, Zhenhui Cao, Hongbin Pan

**Affiliations:** ^1^ Yunnan Provincial Key Laboratory of Animal Nutrition and Feed Science, Faculty of Animal Science and Technology, Yunnan Agricultural University, Kunming, China; ^2^ Institiute of Animal husbandry, Yunnan Vocational and Technical College of Agriculture, Kunming, China; ^3^ Jiangsu Key Laboratory for Molecular and Medical Biotechnology, College of Life Sciences, Nanjing Normal University, Nanjing, China; ^4^ College of Veterinary Medicine, Yunnan Agricultural University, Kunming, China

**Keywords:** antibiotic resistance genes, landrace piglets, saba piglets, diannan small-ear piglets, dahe piglets, jejunum microbiota, different areas

## Abstract

Antibiotic resistance genes (ARGs) are emerging environmental contaminants that threaten human and animal health. Intestinal microbiota may be an important ARGs repository, and intensive animal farming is a likely contributor to the environmental burden of ARGs. Using metagenomic sequencing, we investigated the structure, function, and drug resistance of the jejunal microbial community in Landrace (LA, Kunming), Saba (SB, Kunming), Dahe (DH, Qujing), and Diannan small-ear piglets (DS, Xishuangbanna) from different areas in Yunnan Province, China. Remarkable differences in jejunal microbial diversity among the different pig breeds, while the microbial composition of pig breeds in close areas tends to be similar. Functional analysis showed that there were abundant metabolic pathways and carbohydrate enzymes in all samples. In total, 32,487 ARGs were detected in all samples, which showed resistance to 38 categories of drugs. The abundance of ARGs in jejunum was not significantly different between LA and SB from the same area, but significantly different between DS, DH and LA or SB from different areas. Therefore, the abundance of ARGs was little affected by pig breeds and microorganism community structure, but it was closely related to geographical location. In addition, as a probiotic, *Lactobacillus amylovorus* is also an important ARGs producing bacterium. Our results revealed the antibiotic exposure and intestinal microbial resistance of farms in the study areas, which could provide basic knowledge and potential strategies for rational use of antibiotics and reducing the risk of ARGs transmission in animal husbandry.

## Introduction

With the rapid development of the global economy, demand for animal protein (particularly in low- and middle-income countries) continues to grow at an unprecedented rate (Tilman et al., 2011). This growing demand not only requires increased animal husbandry productivity, but also intensifies the dependence on antibiotics for treating and preventing diseases and promoting growth in animal production (Van Boeckel et al., 2019; Moga et al., 2021). At present, the use of antibiotic in livestock accounts for 73% of global consumption (Van Boeckel et al., 2017). Such high antibiotic use will affect the composition of the gastrointestinal microorganisms in animals and induce antimicrobial resistance (AMR) (Kim et al., 2017).

Approximate 20 antibiotics are commonly used in animal production, with tetracycline, sulfonamide, and penicillin showing the highest resistance rates. Studies on antibiotic resistance in *Escherichia coli*, *Campylobacter* spp., *nontyphoidal Salmonella* spp., and *Staphylococcus aureus* from low- and middle-income countries have greatly thrived since 2000. Meanwhile, antibacterial compounds with resistance higher than 50% have increased from 13% to 34% in pigs (Van Boeckel et al., 2019). Increased antibiotic resistance can lead to the evolution of highly resistant strains, emergence of drug resistance genes (Forslund et al., 2013), development of incurable infections, and rise of morbidity, mortality, and economic loss (Gordillo Altamirano and Barr, 2019). Antibiotic resistance genes (ARGs) and antibiotic-resistant bacteria in animals can enter the environment through feces, and can also spread to humans through the food chain, thus posing a serious threat to public health (Skandalis et al., 2021). At present, the situation of antibiotic resistance in the world is very serious, with higher prevalence in China, India, Brazil, Iran, and Turkey (Van Boeckel et al., 2019). As a major pork producer and consumer, China is also one of the largest users of antibiotics for livestock (Zhu et al., 2013; Krishnasamy et al., 2015). In 2013, China consumed 92,700 tons of antibiotics, with 52.2% used in the pig industry (Zhang et al., 2015). Therefore, high abundance of ARGs is often detected in pork, pig manure and their raising environment (Liu et al., 2019; Mencía-Ares et al., 2020; van Gompel et al., 2020).

Jejunal microflora is closely related to host lipid deposition (Li et al., 2019) and amino acid metabolism (Dai et al., 2010), and play its critical roles in mediation of piglet diarrhea and maintenance of immune homeostasis (Jin et al., 2020). At different growth stages, the content of ARGs in pigs is different, among which the content of ARGs in pig manure is the highest (Tong et al., 2021), while the composition and content of antibiotic resistance genes in piglet jejunum are rarely reported. Jejunal microbial diversity in piglets may differ among different breeds (Gao et al., 2020a), more importantly, environment can also affect the intestinal microbial composition of the host (Su et al., 2021). However, whether these factors affect the AMR in jejunal microorganisms remains unclear.

According to Su et al. (2017), antibiotic pollution in Yunnan province is relatively low. However, as a potential growth area of pig production (Ministry of Agriculture, 2016), an uncontrolled increase in antibiotic use with pig raising will have serious consequences. Yunnan Province is located in southwest China, with unique geographical and climatic characteristics and abundant genetic resources of pig breeds. Protecting these native breeds is of great importance for the sustainable development of the pig industry and for global genetic diversity (Zhao et al., 2021). At present, there are no reports on the use of antibiotics in different local pig breeds. The recommended antibiotics for pig breeding in the same area of Yunnan Province are the same. However, the application of antibiotics in different pig breeds at different areas in Yunnan Province has not been reported. Recently, the introduction of metagenomics has greatly expanded our knowledge in the field of microbiology, which help the researchers look deeper into the composition of intestinal microbial communities and better identify ARGs in the intestinal community. Therefore, in the current study, metagenomic sequencing was implemented to analyze jejunal flora and ARGs in 35-day-old Landrace, Saba, Diannan small-ear, and Dahe piglets at different areas in Yunnan, with ARGs in local pigs at different areas further identified for the first time. These results should provide a theoretical basis for sustainable breeding and elimination of antibiotic resistance in pigs.

## Materials and Methods

### Animals and Sample Collection

One commercial and three local pig breeds raised at different areas in Yunnan were selected (**Table 1**), including Landrace piglets (LA, Kunming City), Saba piglets (SB, Kunming City), Diannan small-ear piglets (DS, Jinghong City), and Dahe piglets (DH, Qujing City). Three pregnant sows were selected from each breed and fed with the National Research Council (NRC, 2012) diet without antibiotics. After delivery, all piglets were mother-fed until day 35. Three piglets were randomly selected from each sow and sacrificed, and the chyme of the middle section of the jejunum was collected. Each breed of piglet contains 9 jejunum chyme samples, randomly selected 3 of which and mixed into a sequencing pool, totally 3 sequencing pools, and sequenced and analyzed. Ethics statements: All animal research was approved by the Ethics Committee of Yunnan Agricultural University (approval No.: YNAU 20200022).

**Table 1:** Sample collection.

Breed^1)^	Type	Location	Sample amounts^2)^	Sequencing pools^3)^
LA	Commercial pig breeds	Wuhua District, Kunming	9	3
SB	Local pig breeds	Luquan County, Kunming	9	3
DH	Local pig breeds	Fuyuan County, Qujing City	9	3
DS	Local pig breeds	Jinghong city, Xishuangbanna Dai Autonomous Prefecture	9	3

1) LA, Landrace piglets; SB, Saba piglets; DS, Diannan small-ear piglets; DH, Dahe piglets.

2) All piglets were fed with breast milk for 35 days, 9 piglets of each variety were randomly selected for slaughter to obtain chyme samples in the middle of jejunum.

3) Each breed of piglet contains 9 jejunum chyme samples, randomly selected 3 of which and mixed into a sequencing pool, totally 3 sequencing pools.

### DNA Extraction and Sequencing

Genomic DNA was extracted from all samples using a HiPure Bacterial DNA Kit (Magen, Guangzhou, China) in accordance with the manufacturer’s instructions. DNA quality was detected using Qubit (Thermo Fisher Scientific, Waltham, MA, USA) and Nanodrop (Thermo Fisher Scientific, Waltham, MA, USA). Bacterial genomic DNA was first fragmented to approximate 350 bp by sonication for subsequent library preparation using a NEBNext^®^ ULtra™ DNA Library Prep Kit for Illumina (NEB, USA). Specifically, DNA fragments (300–400 bp long) were amplified and the polymerase chain reaction (PCR) products were purified using the AMPure XP system (Beckman Coulter, CA, USA). Size distribution of the libraries was analyzed using a 2100 Bioanalyzer (Agilent, Santa Clara, CA, USA), and quantification was performed using real-time PCR. Genomic sequencing was carried out on an Illumina HiSeq 4000 sequencer by Gene Novo Biotechnology Co., Ltd. (Guangzhou, China) using paired-end technology (PE 150). The raw reads of metagenome sequencing have been submitted to the NCBI SRA database under the accession PRJNA757176.

### Bioinformatics Analysis and Function Annotations

FASTP (v0.18.0) (Chen et al., 2018) was applied to filter out low quality raw data from the Illumina platform using the following criteria: 1) reads containing ≥10% unidentified nucleotides (N); 2) reads with ≥50% of bases having phred quality scores ≤20; and 3) reads aligned with a barcode adapter. Clean reads obtained after filtering were aligned to the reference genome of the host using Bowtie2. After filtering host reads, effective reads were obtained and used for genome assembly. Effective reads of each sample were assembled individually using MEGAHIT (v1.1.2) (run parameters: -continue -m 60000000000 -t 6 -min-contig-len 500 -presets meta-sensitive) stepping over a k-mer range of 21–99 to generate sample-derived assembly (Li et al., 2015). Metagenemark (v3.38) (run parameters: -a -d -f G –m) was used for gene determination based on the final assembled contig (>500 bp) (Zhu et al., 2010). To reduce the number of redundant genes in the downstream assembly, based on ≥95% identity and 90% read coverage, CD-HIT (v4.6) (run parameters: -aS 0.9 -c 0.95 -g 1) was used to collect and combine genes with lengths ≥300 bp in all samples (Fu et al., 2012). Reads were realigned to the predicted genes using Bowtie (v2.2.5) (bowtie2 -q –mp 1,1 –np 1 –score-min L,0, -0.1 –no-mixed –no-discordant) to count read numbers (Langmead and Salzberg, 2012). The final gene catalog was obtained from nonredundant genes with a gene read count of >2. Gene abundance was calculated using the following formula:


$$G_{k}=\frac{r_{k}*10^{9}}{L_{k}*\sum_{i=1}^{n}r_{i}}$$

where r is the number of reads of the aligned genes and L is the length of the genes.

Using DIAMOND software (v0.9.24, threshold value <=1e-5) (Buchfink et al., 2015), unigenes were annotated based on the National Center for Biotechnology Information (NCBI) non-redundant protein database, Kyoto Encyclopedia of Genes and Genomes (KEGG), Evolutionary Genealogy of Genes: Non-Supervised Orthologous Groups (eggNOG), Carbohydrate-Active enZYmes (CAZy), and Comprehensive Antibiotic Resistance Database (CARD). We used Kaiju (v1.6.3) (run parameters: -a greedy –e 3 –s 65) to translate the effective reads into amino acid sequences and disconnect them at the terminator (Menzel et al., 2016). The target amino acid sequences were then screened using the Greedy model based on a score >65 and compared to the NCBI RefSeq database (v20190205) to obtain the species classification characteristics of the sequences and calculate species abundance in each sample at different classification levels.

### Statistical and Data Analysis

One-way ANOVA is used to analyze the abundance of antibiotic resistance genes of different types in different pig breeds and it was performed using SPSS (v21), correlation analysis between differential microorganisms and different categories ARGs in jejunum was estimated using Spearman test, and *p* < 0.05 was considered significant. A Venn graph was plotted using the Venn diagram package in the R project (Chen and Boutros, 2011). Principal coordinate analysis (PCoA) was calculated using the vegan package and plotted using the ggplot2 package in R (v0.7). Violin plots were plotted using the ggplot2 package (v0.7). Statistical analysis of similarities (ANOSIM) was performed using the vegan package (v1.17-4). Circular layout representations of species or functional gene abundance were graphed using Circos (v0.69-3). Biomarker features in each group were screened using LEfSe (v1.0) (Segata et al., 2011). Stack column, pie chart and heatmap were generated using GraphPad Prism (v7.0).

## Results

### Summary of the Metagenomic Datasets

**Figure 1:**
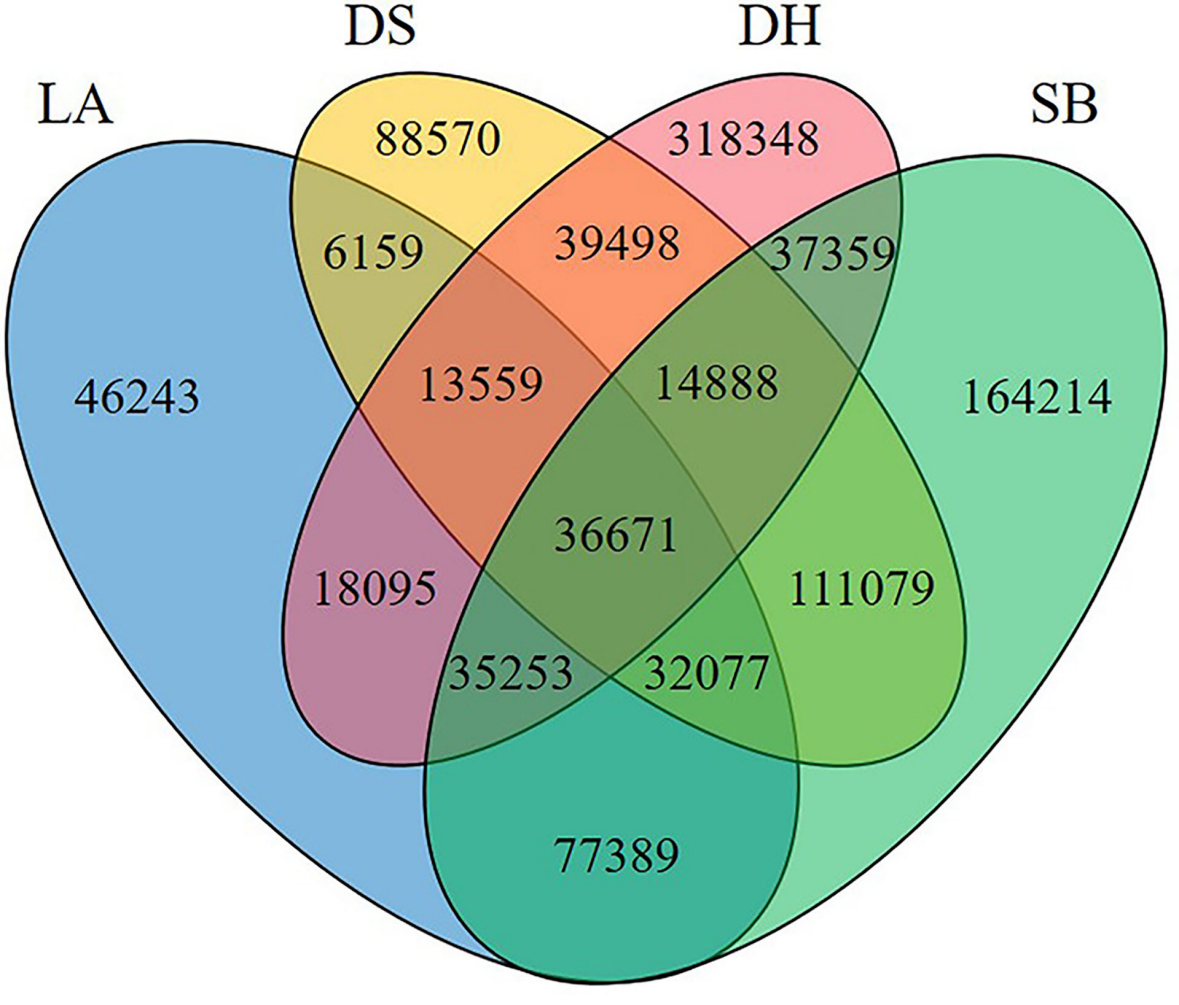
Number of common and unique genes among different populations. LA (blue), Landrace piglets, SB (green), Saba piglets, DS (yellow), Diannan small-ear piglets, DH (red), Dahe piglets.

Jejunal chyme samples from four pig breeds were analyzed by metagenomic sequencing, with 974,488,766 raw reads obtained, and there was no significant difference among LA (842,039,78.00 ± 117,218,87.33), SB (958,454,62.67 ± 729,828,9.29), DS (739,609,66.67 ± 528 297 6.29) and DH (708,191,81.33 ± 402,735,8.99) groups. After filtering and assembling these reads, a total of 1,284,875 contigs (>500 bp) were obtained. 2,913,711 unigenes were predicted from contigs >500 bp, in which the number of unigenes in DH (401,751.33 ± 611,2.69) group was significantly higher than that in LA (144,176.33 ± 264,36.65), SB (240,571.00 ± 326,27.38) and DS (184,738.33 ± 492,61.16) groups (*p* = 0.002) (**Supplementary Table 1**). A Venn graph (**Figure 1**) illustrated the number of shared and unique genes among the four groups, with 36,671 genes common in all four groups. The number of genes unique to LA, SB, DS, and DH was 46,243, 164,214, 88,570, and 318,348, respectively.

### Comparison of the Jejunal Microbial Composition

The pig jejunum harbors an extremely complex and dynamic microbial community, including fungi, bacteria, viruses, and archaea. Our results showed that more than 80% of the microbial community consisted of bacteria. Firmicutes, Proteobacteria, Bacteroidetes, and Actinobacteria were the four most dominant phyla in the four pig breeds, while Firmicutes was the most abundant bacterial taxa in all samples, accounting for 60.54%–85.43% of the total bacterial community. In addition, the relative abundance of Bacteroides in Saba Pig (SB) (2.45 ± 0.80%), Diannan small-ear pig (DS) (1.94 ± 0.90%) and Dahe (DH) (14.54 ± 0.11%) is higher than that in Landrace Pig (LA) (1.08 ± 0.13%) (**Supplementary Table 2**).

**Figure 2:**
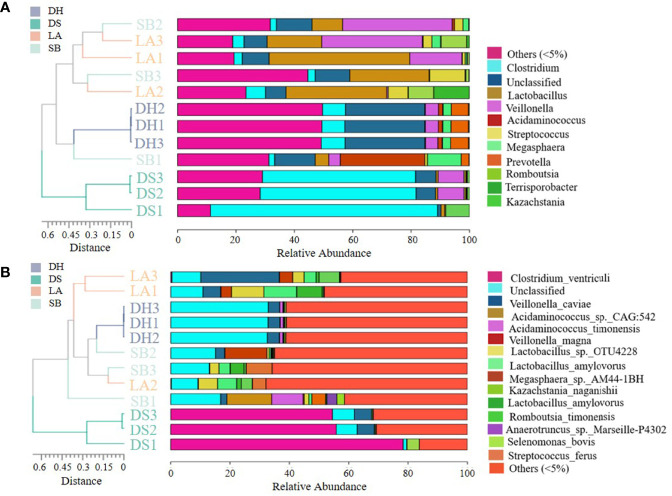
Unweighted pair-group method with arithmetic mean (UPGMA) clustering tree. Relative abundance and similarity of bacterial communities with abundance greater than 5% at genus **(A)** and species **(B)** level. Sequences that could not be classified into any known group were grouped as “unclassified”, and sequences detected with low abundance were grouped as “others”.

**Figure 3:**
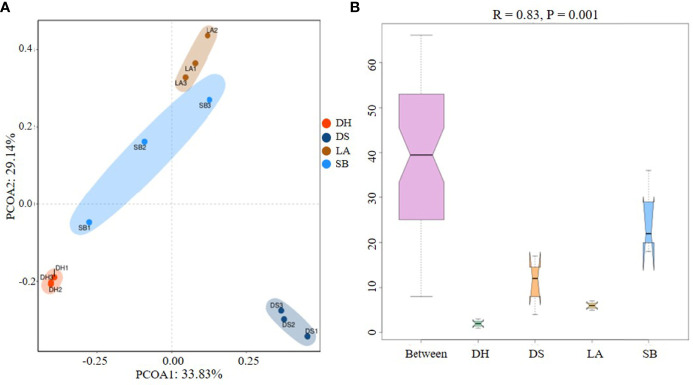
**(A)** Principal coordinate analysis (PCoA) of intestinal microbes among different pig breeds. Community composition of pig breeds differed at species level, with 33.83% for PCO1 and 29.14% for PCO2. **(B)** Analysis of similarity (ANOSIM) plots for variations in four groups. Between (all samples), LA, SB, DS, and DH on X-axis represent different groups and numbers on Y-axis represent UniFrac rank. Differences between groups were higher than differences within groups when Between group rank was higher than that of other groups. R-value ranged from -1 to 1, with R > 0 indicating higher intergroup differences than intragroup differences.

Cluster analysis at genus and species level showed that all samples were divided into two different clusters. The first cluster included LA, SB and DH, while the second cluster included DS (**Figure 2**); At the species level, PCoA plot clearly separated the 12 samples based on the pig breeds (**Figure 3A**), indicating distinct differences in bacterial community structure among the four pig breeds. Furthermore, the results of PCoA were confirmed by the ANOSIM diagram (**Figure 3B**), which showed that the variation between different pig breeds was significantly greater (R = 0.83, *p* = 0.001) than that within the same pig breed. Difference between groups was higher than that within groups, as the rank for between group was higher than those for the other groups. Interestingly, the intestinal microbial composition in LA, SB and DH pigs from close areas tended to be similar, independent of the genetic background of the host.

### Functional Annotation

**Figure 4:**
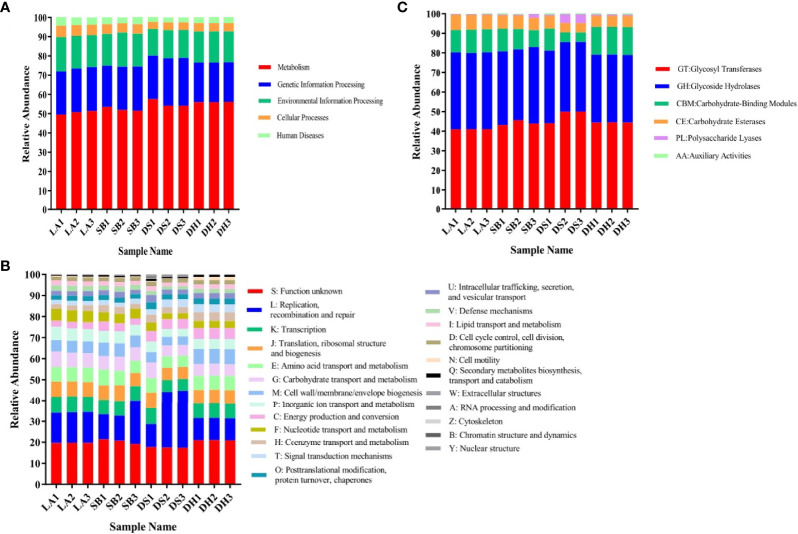
Relative abundance based on level A hits in **(A)** KEGG, **(B)** eggNOG, and **(C)** CAZy functional categories in each sample.

To reveal the potential functional capacity of the porcine jejunal microbiota, unigenes were annotated to the KEGG, eggNOG, and CAZy databases. Approximately 61.20%, 66.21% and 10.03% of genes were assigned to KEGG pathways, eggNOG database and CAZy database (**Supplementary Table 3**), respectively. Functional abundance graphs of each sample based on the relative abundance of level A hits in the three databases were plotted (**Figure 4**).

KEGG pathway analysis indicated that all samples were enriched in metabolic pathways, genetic information processing, environmental information processing, cellular processes, and human diseases, among which metabolic pathways were the most abundant (**Figure 4A**). EggNOG analysis (**Figure 4B**) also identified genetic information processing, carbohydrate transportation, and metabolism as the most abundant pathways, thus confirming the KEGG results. Carbohydrate-active enzymes encoded by intestinal microorganisms play a crucial role in host digestion and absorption of complex dietary carbohydrates (Wang et al., 2019). CAZy analysis revealed that glycosyl transferase (GT), glycoside hydrolases (GH), carbohydrate-binding modules (CBM), and carbohydrate esterases (CE) were abundant in all samples (**Figure 4C**), which may be related to the strong digestion and absorption ability of pigs.

### LEfSe Analyses of Differentially Abundant Functions

LEfSe analysis was used to identify high-dimensional biomarkers in each group (LDA score [log10] > 2). The LEfSe analysis diagrams (**Supplementary Figures S1–S3**) showed that significant enrichment of 16 KEGG pathways, 3 orthologous groups, and 17 carbohydrate enzymes were observed in LA pigs; while 6 KEGG pathways and 5 carbohydrate enzymes were enriched in SB pigs; 25 KEGG pathways, 2 orthologous groups, and 14 carbohydrate enzymes were significantly enriched in DS pigs; and 24 KEGG pathways, 7 orthologous groups, and 57 carbohydrate enzymes were significantly enriched in DH pigs.

Based on LEfSe analysis of the KEGG, eggNOG, and CAZy pathways (**Supplementary Figures S1–S3**), the top differentially abundant pathways among the four breeds are summarized in **Table 2**. Results showed that KEGG orthologs such as starch and sucrose metabolism (Ko00500), phosphotransferase system (Ko02060), and quorum sensing (Ko02024) were significantly enriched in LA. ABC transporters (Ko02010), glycerolipid metabolism (Ko00561), and riboflavin metabolism (Ko00740) were significantly enriched in SB. ribosome (Ko03010), ascorbate and aldarate metabolism (Ko00053), and galactose metabolism (Ko00052) were significantly enriched in DS. and phenylalanine, tyrosine, and tryptophan biosynthesis (ko00400), two-component system (Ko02020), and homologous recombination (ko03440) were significantly enriched in DH.

**Table 2:** Top differentially abundant pathways among the four pig breeds.

Pathway/Enzyme	LA	SB	DS	DH
	Ko00500 (Starch and sucrose metabolism)	Ko02010 (ABC transporters)	Ko03010 (Ribosome)	Ko00400 (Phenylalanine, tyrosine, and tryptophan biosynthesis)
KEGG	Ko02060 (Phosphotransferase system)	Ko00561 (Glycerolipid metabolism)	Ko00053 (Ascorbate and aldarate metabolism)	Ko02020 (Two-component system)
	Ko02024 (Quorum sensing)	Ko00740 (Riboflavin metabolism)	Ko00052 (Galactose metabolism)	Ko03440 (Homologous recombination)
	F: Nucleotide transport and metabolism		Y: Nuclear structure	N: Cell motility
eggNOG	P: Inorganic ion transport and metabolism		B: Chromatin structure and dynamics	S: Function unknown
	V: Defense mechanisms			M: Cell wall/membrane/ envelope biogenesis
	GH73	GT24	GT1	GH3
CAZy	CE14	GT32	GH38	GT9
	CE12	GH68	GT47	GH31

LA, Landrace piglets; SB, Saba piglets; DS, Diannan small-ear piglets; DH, Dahe piglets.

Alignment of the metagenomic data in the eggNOG database revealed that nuclear transport and metabolism, organic transport and metabolism, and defense mechanisms were highly enriched in the LA. nuclear structure and chromatographic structure and dynamics were significantly enriched in the DS. cell motility and cell wall/membrane/envelope biogenesis were significantly enriched in the DH, while no significant enrichment was observed in the SB pigs.

Similarly, the metagenomic data were aligned to the CAZy database, which showed that GH73, CE14, and CE12 were higher in the LA pigs. GT24, GT32, and GH68 were higher in the SB pigs. GT1, GH38, and GT47 were higher in the DS pigs, and GH3, GT9, and GH31 were higher in the DH pigs.

### Antibiotic Resistance Profiles

**Figure 5:**
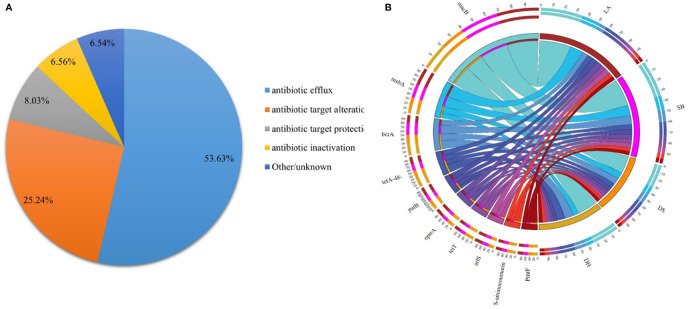
Resistance Mechanism and antibiotic resistance ontologies of antibiotic resistance genes. **(A)** Composition and proportion of resistance mechanisms in four pig breeds. **(B)** Functional distribution of top 10 most abundant antibiotic resistance ontologies in each group.

To evaluate whether some breeds or environments contribute to the maintenance and spread of ARGs, the predicted gene sequences were annotated based on CARD. A total of 32,487 ARGs was detected in all samples, accounting for 3.13% of all genes. Each gene was annotated with information on resistance mechanisms and drug classes (**Supplementary Table 3**). The main mechanisms of drug resistance in the detected drug resistant genes included antibiotic efflux (53.63%), antibiotic target alteration (25.24%), antibiotic target protection (8.03%), and antibiotic inactivation (6.56%) (**Figure 5A**). All ARGs were grouped into 720 antibiotic resistance ontologies (AROs) (**Supplementary Table 3**). The top 10 most abundant AROs were shown in **Figure 5B**. Furthermore, macB, msbA and bcrA are the most contributing AROs in all the samples, and macB was most prevalent one.

**Figure 6:**
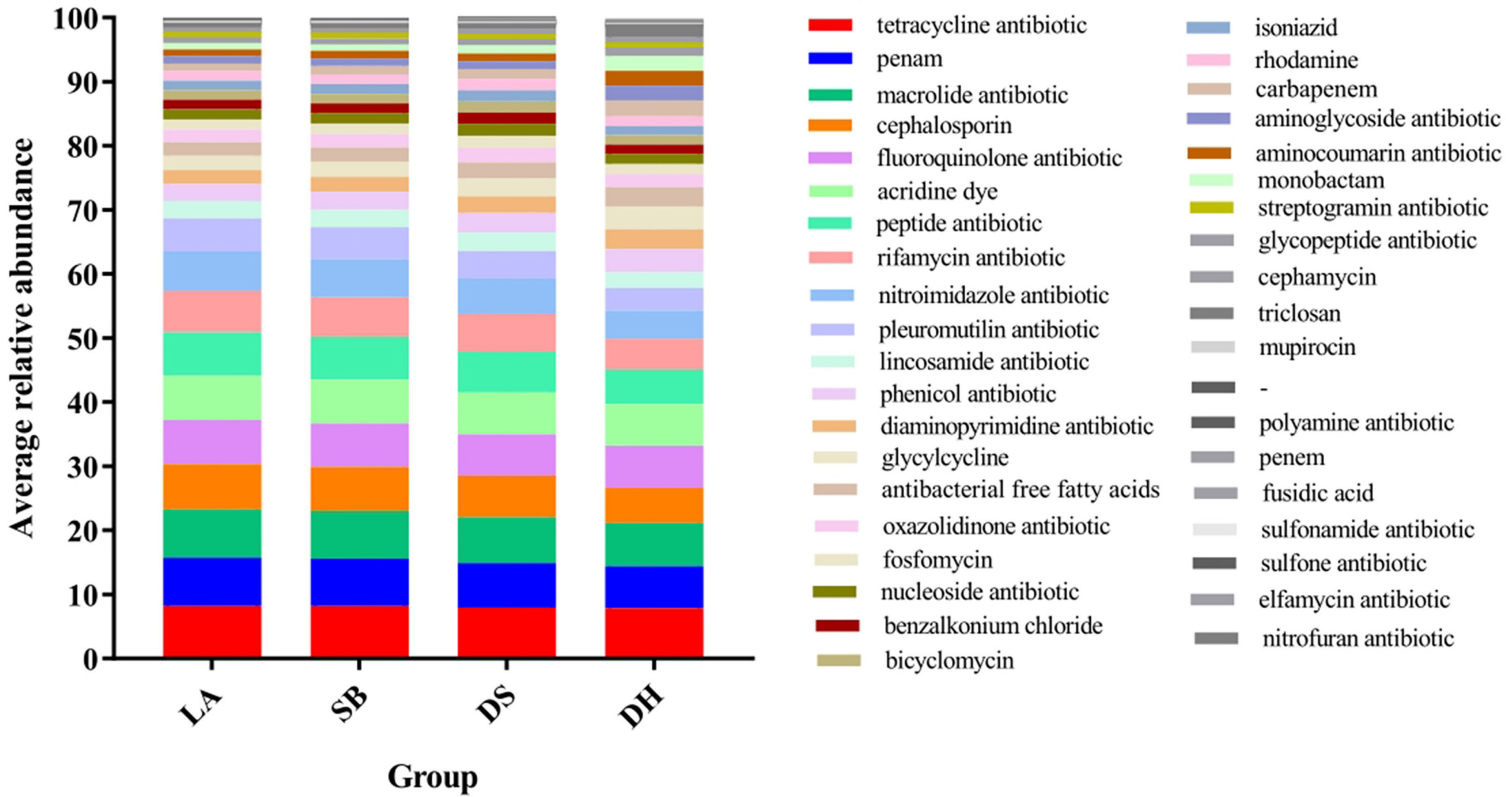
Stack column of average relative abundance of antibiotic resistance genes in different categories among pig breeds.

ARG types were matched with their corresponding antibiotics and the average relative abundance of the genes conferring antibiotic resistance in the different pig breeds were summarized (**Figure 6**). All samples had ARGs from the same class of antibiotics. Resistance to 38 categories of drugs, including almost all major antibiotics used in clinics and agriculture, was detected in the intestinal microbes. The top 10 drug categories with ARG abundance in all samples included tetracycline, macrolide, penicillin, fluoroquinolone, acridine dye, cephalosporin, peptide, rifamycin, nitroimidazole, and pleuromutilin, while resistance to the remaining antibiotic classes was relatively low. The relative abundance of these 10 ARG types were compared (**Table 3**), it was found that they were all significantly higher in LA and SB pigs than in DS pigs (*p* < 0.05). The abundance of ARGs against penicillin, cephalosporins, peptides, rifamycin, nitroimidazole, and pleuromutilin in LA pigs, and against cephalosporins, peptides, rifamycin, nitroimidazole, and pleuromutilin in SB, were significantly higher than that in DH (*p* < 0.05). The abundance of ARGs against fluoroquinolones and nitroimidazoles in DH was significantly higher than that in DS (*p* < 0.05). Thus, these results indicate significant differences in ARG abundance in pig breeds at different areas, but no significant differences at same area.

**Table 3:** Comparison of abundance of the different types of antibiotic resistance genes.

	LA	SB	DS	DH	*p*-value
**Tetracycline antibiotic**	**41 620.37 ± 900.72^a^**	**40 818.83 ± 2 640.79^a^**	**31 240.24 ± 3 438.64^b^**	**37 483.35 ± 24.79^ab^**	**0.039**
**Macrolide antibiotic**	**38 340.56 ± 940.44^a^**	**37 089.90 ± 2 416.73^a^**	**28 345.95 ± 2 834.27^b^**	**32 315.43 ± 7.56^ab^**	**0.022**
**Penam**	**38 207 ± 1 103.20^a^**	**36 819.68 ± 2 318.63^ab^**	**27 335.89 ± 2 699.05^c^**	**31 476.57 ± 25.25^bc^**	**0.011**
**Fluoroquinolone antibiotic**	**35 032.23 ± 839.05^a^**	**34 003.55 ± 2 167.37^a^**	**25 585.43 ± 2 520.91^b^**	**31 398.96 ± 17.28^a^**	**0.018**
**Acridine dye**	**35 030.17 ± 840.12^a^**	**33 993.26 ± 2 169.73^a^**	**25 582.61 ± 2 522.05^b^**	**31 397.31 ± 16.71^a^**	**0.018**
**Cephalosporin**	**36 023.86 ± 1 057.43^a^**	**34 498.37 ± 2 167.39^a^**	**25 469.45 ± 2 709.19^b^**	**26 328.95 ± 44.72^b^**	**0.006**
**Peptide antibiotic**	**34 494.84 ± 957.13^a^**	**33 083.35 ± 2 011.03^a^**	**24 998.86 ± 2 516.81^b^**	**25 643.73 ± 22.61^b^**	**0.007**
**Rifamycin antibiotic**	**32 881.66 ± 933.75^a^**	**31 160.67 ± 2 174.91^a^**	**23 344.82 ± 2 352.97^b^**	**23 063.36 ± 36.21^b^**	**0.005**
**Nitroimidazole antibiotic**	**31 271.88 ± 962.91^a^**	**29 548.39 ± 2 043.94^a^**	**21 682.21 ± 2 323.41^b^**	**21 320.92 ± 35.93^b^**	**0.004**
**Pleuromutilin antibiotic**	**26 247.69 ± 1 013.98^a^**	**24 767.33 ± 1 932.25^a^**	**16 890.5 ± 1 557.79^b^**	**16 739.05 ± 16.11^b^**	**0.001**

one-way ANOVA was used for statistical analysis. The results are expressed by mean values ± standard error of the mean, and "a,b,c,ab,bc" means significant difference or not. In same row, containing the same superscript indicates no significant difference (p > 0.05), different superscript indicates significant difference (p < 0.05). LA, Landrace piglets; SB, Saba piglets; DS, Diannan small-ear piglets; DH, Dahe piglets.

### Correlation Analysis Between Differential Microorganisms and Different Categories ARGs

**Figure 7:**
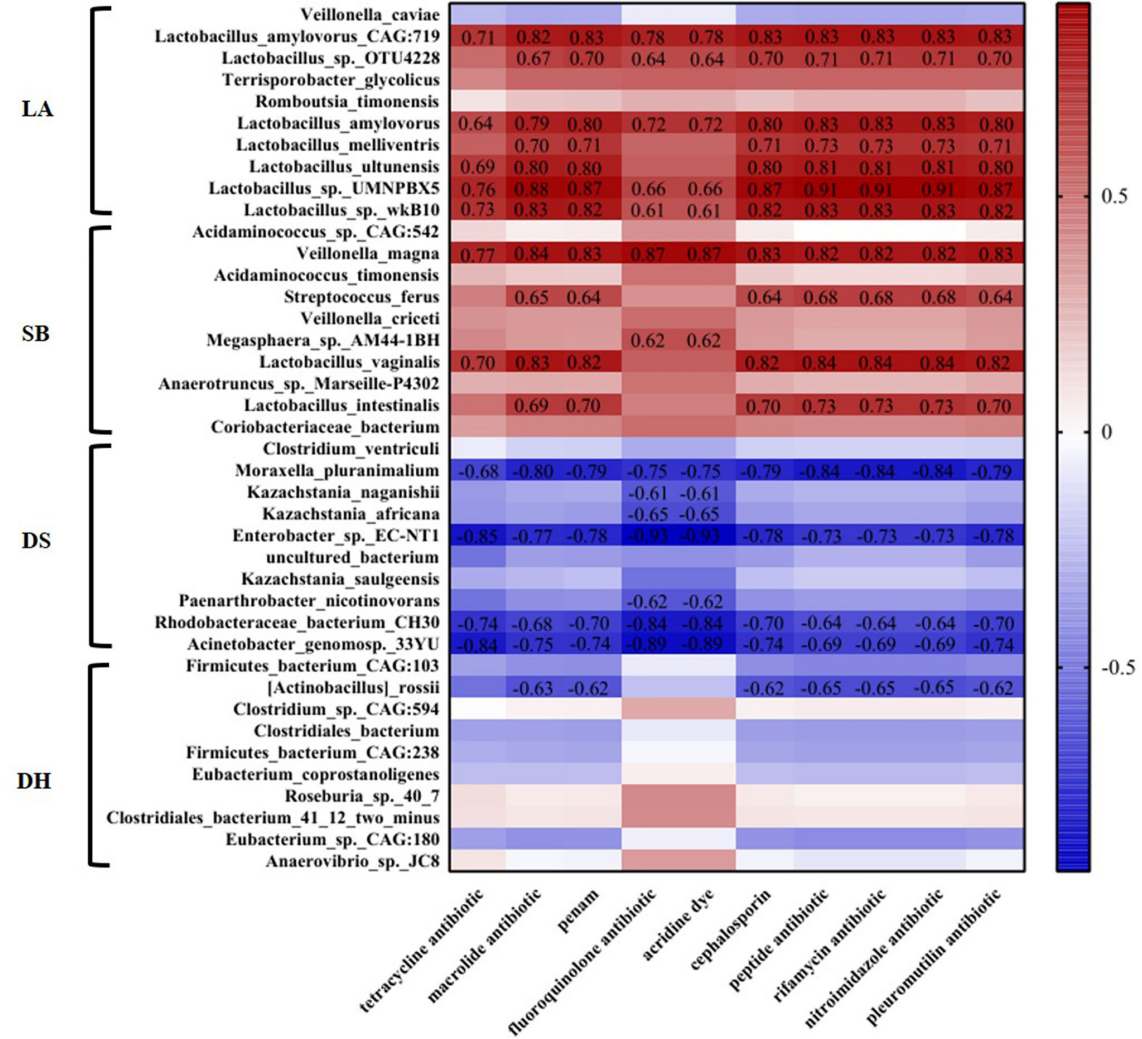
Heatmap analysis of the abundance of top ten differential microorganisms and different categories ARGs in different pig breeds. Correlation of *p* < 0.05 are shown. The number in the figure represents R value.

Next, we analyzed the correlation between the abundance of top ten differential microorganisms and different categories ARGs in different pigs by Spearman’s correlation heatmap. It was found that 20 species of microorganisms were significantly correlated with ARGs. Among them, 12 species of microorganisms with positive correlation with ARGs belonged to Firmicutes, and they had the highest abundance in LA or SB, and 9 of them belonged to *Lactobacillus*. Specifically, the positive correlation with tetracyclines resistance gene were *Lactobacillus amylovorus* CAG:719 (*p* = 0.012, R = 0.71), *Lactobacillus amylovorus* (*p* = 0.028, R = 0.64), *Lactobacillus ultunensis* (*p* = 0.016, R = 0.69), *Lactobacillus* sp. UMNPBX5 (*p* = 0.0055, R = 0.76), *Lactobacillus* sp. wkB10 (*p* = 0.0096, R = 0.73) and *Lactobacillus vaginalis* (*p* = 0.014, R = 0.70), the positive correlation with macrolides, penicillin, cephalosporin, peptide, rifamycin, nitroimidazole and pleuromutilin resistance genes were *Lactobacillus amylovorus* CAG:719 (*p <*0.01, R >0.81), *Lactobacillus* sp. OTU4228 (*p <*0.05, R >0.67), *Lactobacillus amylovorus* (*p <*0.01, R >0.79), *Lactobacillus melliventris* (*p <*0.05, R >0.70), *Lactobacillus ultunensis* (*p <*0.01, R >0.79), *Lactobacillus* sp. UMNPBX5 (*p <*0.01, R >0.87), *Lactobacillus* sp. wkB10 (*p <*0.01, R >0.81), *Lactobacillus vaginalis* (*p <*0.01, R >0.81) and *Lactobacillus intestinalis* (p <0.05, R >0.69), and the positive correlation with fluoroquinolone and acridine dye resistance genes were *Lactobacillus amylovorus* CAG:719 (*p* = 0.0038, R = 0.78, *p* = 0.0038, R = 0.78), *Lactobacillus* sp. OTU4228 (*p* = 0.028, R = 0.64, *p* = 0.028, R = 0.64), *Lactobacillus amylovorus* (*p* = 0.011, R = 0.72, *p* = 0.011, R = 0.72), *Lactobacillus* sp. UMNPBX5 (*p* = 0.022, R = 0.66, *p* = 0.022, R = 0.66) and *Lactobacillus* sp. wkB10 (*p* = 0.040, R = 0.61, *p* = 0.040, R = 0.61). The remaining 8 microorganisms negatively correlated with ARGs were distributed in the three Phyla of Proteobacteria, Ascomycota and Actinobacteria (**Figure 7**).

## Discussion

The intestinal tract of mammals contains many microorganisms, which exert essential effects on metabolism, nutrient absorption, and immune function of the host (Walsh et al., 2014). Functional heterogeneity of each gastrointestinal tract segment gives rise to regional differences in gut microbial populations (Martinez-Guryn et al., 2019). The interaction among intestinal microflora, intestinal mucosa and nutrient flow is an important way for the body to absorb nutrients, and jejunum is the main region of nutrient absorption (El Aidy et al., 2013). In addition, jejunal microbiota participates in the regulation of immune response and mediates piglet diarrhea (Bin et al., 2018; Jin et al., 2020).

The population structure of gut microbiota in vertebrates is regulated by both environmental and genetic factors of the host (Zhao et al., 2013). Previous studies have shown that there were significant differences in intestinal microbial composition among different breeds of pigs (Yang et al., 2014; Li et al., 2020). In our study, PCoA and ANOSIM analysis clearly showed distinct population structure at species level among four pig breeds, which confirmed the differences of intestinal microbes among different pig breeds. In addition, the environment plays a great role in determining the composition of intestinal microflora (Rothschild et al., 2018). β-diversity analysis revealed significant differences in fecal microbial composition among Tibetans (Lan et al., 2017), yaks (Fan et al., 2020), and wild sables (Su et al., 2021) at different altitudes, but similar bacterial population structure in European mouflon and blue sheep samples at the same altitude (Sun et al., 2019). This is consistent with our result, cluster analysis showed that the intestinal flora of LA, SB and DH in geographically close areas tends to be similar (Kunming was 147 kilometers away from Qujing), but they were obviously separated from DS located in far away areas (Jinghong was 692 kilometers away from Kunming and 664 kilometers away from Qujing). These results indicate that the change of intestinal microbial composition may be an important factor for pigs to adapt to different environments.

Intestinal microflora has strong metabolic potential *via* extraction of energy from host indigestible carbohydrates (dietary fiber), transformation of host-derived substances and provision of an important source of nutrition for the host (Blaut, 2013). The overall analyses of microbial function in this study indicated that the intestinal microorganisms of pigs had high metabolic activity and may participate in many functional metabolic pathways, such as amino acid, carbohydrate and inorganic ion metabolisms. These diverse and enriched metabolic pathways may be related to the high energy consumption required for pig growth.

ARGs are environmental pollutants that pose potential risks to human and animal health (Petrin et al., 2019). The level of antibiotic resistance can vary between regions and countries, corresponding to the degree of antibiotic consumption (Benbachir et al., 2001). An AMR survey of EU member states found that antibiotic resistance is higher in southern and southeastern countries than in northern countries (Ferri et al., 2017). Liu et al. (2018) show that normalized ARGs abundance in Chinese waterbodies is higher in southern/central China than in northern China (northwest and northeast). In addition, oral and non-oral *Enterococcus faecalis* bacteria in Japan, Britain, and Brazil (Lins et al., 2019), microbial isolates from patients with eye infections in western America (Asbell et al., 2020) and *Vibrio cholerae* O1 and *Shigella* species isolates in Bangladesh (Klontz et al., 2014) all exhibit geographically heterogeneous drug resistance. Moreover, Cao et al. (2020) found that host resistance was independent of its microorganism community structure. This is consistent with our research, we detected high levels of ARGs in pig breeds in Kunming. At the same area, there is no significant difference between LA and SB, but there are significant differences among varieties at different areas. Kunming, as the capital city of Yunnan Province, has relatively dense and developed animal husbandry, followed by Chuxiong, and Jinghong is the most backward. Therefore, ARGs abundance appears to be independent of pig breed and microorganism community structure but closely related to its living environment and animal husbandry development. What’s more, our results may reflect differences in exposure to antibiotics in different areas.

Antibiotic overuse, whether to control bacterial diseases or as a performance enhancer, will promote the development of AMR (van den Bogaard and Stobberingh, 1999). In low- and middle-income countries, high resistance to widely used antibiotics has been found in common pathogenic bacteria in animal breeding, including tetracyclines, sulfonamides, penicillin, quinolones, macrolides, and cephalosporins (Van Boeckel et al., 2019). A study on antibiotic use in poultry in eight countries, including China, USA, and France, shows high drug resistance rates to tetracyclines, aminoglycosides, sulfonamides, and penicillin (Roth et al., 2019). In addition, high abundance of resistance genes to tetracyclines, macrolides, β-lactams, aminoglycosides, and sulfonamides has been reported in the intestinal microflora of pigs and cattle in Korea (Lim et al., 2020). Tetracyclines are widely used broad-spectrum antibiotics in animal husbandry (Griffin et al., 2011; Chang et al., 2014). Previous studies demonstrated high residue of tetracycline resistance genes (TCs) in livestock and poultry manure (Tian et al., 2021), with higher content in pig feces than other veterinary antibiotics (Li et al., 2013; Zhi et al., 2020). Macrolides possess antibacterial, anti-inflammatory, and immunomodulatory properties (Zhuo et al., 2014). These antibiotics are widely used in human and veterinary medicine (Wang, 2009), and are also common environmental pollutants in water (Galvidis et al., 2015). Penicillin is broadly used in many food animals and is also one of the most important drug residues in animal-derived foods (Li et al., 2017). In our study, the resistance trends of tetracyclines, macrolides, and penicillin in the four pig breeds are consistent with previous studies, but cephalosporins were the antibiotics with the fourth resistance rate in LA and SB, and fluoroquinolones were the antibiotics with the fourth resistance rate in DS and DH, which indicates that there are some differences in the use of antibiotics of pig breeds in different regions in the investigated areas. We also detected low resistance rates to aminoglycosides and sulfonamides in the four pig breeds, inconsistent with previous reports (Roth et al., 2019; Lim et al., 2020), which may be due to different antibiotic used in different areas.

Bacteria may be resistant to one or more antimicrobials, and different bacteria have different contributions to the generation of ARGs, and bacteria of Firmicutes are the main sources of ARGs of greylag geese and ruddy shelducks (Wang et al., 2019). This is similar to our research results, that is, among the top ten abundant microbes with differences in each pig breed, there were 12 species of microorganisms that were significantly positive correlated with ARGs, and they all belong to Firmicutes, which indicates that Firmicutes microorganisms are important ARGs-producing bacteria. Besides, these microorganisms had the highest abundance in LA or SB, which is consistent with the trend of ARGs abundance in different areas, so the difference of these microorganisms may be the reason for the difference of ARGs abundance among different pig breeds. Common resistance mechanisms of bacteria include: 1. Mutation of antimicrobial target site, usually to change the binding target of antimicrobials to reduce their binding efficiency, such as macrolide drugs, when the 23S rRNA gene has point mutation, its therapeutic effect will become worse. 2. Enzymatic degradation or alteration of antibacterial, as β-lactamases through cleaves the β-lactam ring enzymatically, leading to the failure of β-lactam drugs. 3. Reduce drug accumulation, for instance bacteria gain tetracycline resistance through energy-dependent efflux proteins encoded by tet genes (Chopra and Roberts, 2001; Wright, 2005; Harbottle et al., 2006; Robicsek et al., 2006; Flórez et al., 2007). In this study, there are 12 species of microorganisms that are positively correlated with ARGs. These microorganisms may be resistant to antibiotics through one or more of the above mechanisms. Among them, nine species of microorganisms belong to *Lactobacillus*. *Lactobacillus* belongs to Firmicutes, Gram-positive bacterium, which is a part of animal intestinal symbiotic flora, plays an important role in the balance of host microorganisms, and is also a kind of probiotics widely used in food (Lebeer et al., 2008). However, with the development of antibiotic resistance, the prevalence of ARGs has been found in *Lactobacillus* in animal intestines and feces (Dec et al., 2017; Dec et al., 2018). It is reported that the prophage has a profound influence on the host bacteria, and the ARGs produced during its integration, release, and dissemination are related to the phenotypic resistance of the host bacteria. Almost all lactobacilli contain prophage fragments, and more than 10% of the prophage have ARGs (Pei et al., 2021). Therefore, this may be an important reason why lactobacilli become ARGs carriers. In our study, *Lactobacillus* was significantly positive correlated with multiple ARGs, which indicates that *Lactobacillus* in animal intestines is a reservoir of ARGs. At the species level, Campedelli et al. (2018) reported *Lactobacillus amylovorus*, *Lactobacillus melliventris*, *Lactobacillus ultunensis*, *Lactobacillus vaginalis* and *Lactobacillus intestinalis* of animal origin are all resistant to macrolide, penicillin and rifamycin, which is consistent with our research, indicated that these microbes in animals are common antibiotic-producing bacteria. Besides, Sun et al. (2021) found that the resistance of dairy cows to tetracenomycin C shows a high prevalence rate, while *Lactobacillus amylovorus* has a strongest association with resistance to tetracenomycin C. *Lactobacillus amylovorus* is a beneficial microbiota present in the intestines of piglets (Kant et al., 2011), which can be used to treat diarrhea of piglets, respiratory diseases of dairy cows and enhance gastrointestinal immunity of animals (Roselli et al., 2007; Martinez et al., 2013; Amat et al., 2020). Our results showed that the resistance of *Lactobacillus amylovorus* to ten categories ARGs was positively correlated and its relative abundance was significantly higher in LA. When *Lactobacillus* probiotics are isolated from the intestines of piglets, our results will provide basic knowledge and potential strategies for reducing the risk of ARGs transmission.

It is found that livestock breeding will lead to the groundwater around the farm and even the living well water of nearby residents being polluted by veterinary antibiotics and ARGs, thus increasing the potential health risks of local residents (Gao et al., 2020b). Antibiotic abuse has likely led to the emergence and expansion of ARGs. Therefore, the use of antibiotics should be reduced (or eliminated) in both humans and animals. With the rapid development of the global economy, animal production is gradually increasing. By implementing strict hygiene standards for newly built farms, it may be possible to restrict the development of AMR. Such practices can reduce the risk of drug-resistant pathogen transmission, especially in areas where veterinary antibacterial agents promote intensive meat production (Van Boeckel et al., 2017).

## Conclusions

This study provided metagenomic resources for the jejunum flora of four pig breeds raised at different areas in Yunnan Province, China. Our result demonstrated that there were remarkable differences in jejunal microbial diversity among the different pig breeds, while the microbial composition of pig breeds in close areas tends to be similar. In addition, our ARG analyses showed that *Lactobacillus* is an important ARGs producing bacterium, and the abundance of ARGs was little affected by pig breeds and microorganism community structure, but it was closely related to geographical location. These data may reflect the antibiotic exposure at different regions in Yunnan Province, and provide a reference for the rational use of antibiotics.

## Data Availability Statement

The datasets presented in this study can be found in online repositories. The names of the repository/repositories and accession number(s) can be found in the article/**Supplementary Material**.

## Ethics Statement

The animal study was reviewed and approved by All animal research was approved by the Ethics Committee of Yunnan Agricultural University (approval No.: YNAU 20200022). Written informed consent was obtained from the owners for the participation of their animals in this study.

## Author Contributions

HP designed the experiments. HP, YL, YY, LM, ZC, QA, CZ and GY performed the experiments. YL and YY analyzed the data and wrote the manuscript. JL and ZC revised this manuscript. All the authors contributed to the article and approved the submitted version.

## Funding

This study was supported by National Natural Science Foundation of China (NOs. 32160795 and U1802234).

## Conflict of Interest

The authors declare that the research was conducted in the absence of any commercial or financial relationships that could be construed as a potential conflict of interest.

## Publisher’s Note

All claims expressed in this article are solely those of the authors and do not necessarily represent those of their affiliated organizations, or those of the publisher, the editors and the reviewers. Any product that may be evaluated in this article, or claim that may be made by its manufacturer, is not guaranteed or endorsed by the publisher.

## Supplementary Material

Supplementary Figure 1 The LEfSe analysis was used to find high-dimensional biomarkers in jejunum microorganisms of different pig breeds. Functional abundance through KEGG pathways.

Supplementary Figure 2 The LEfSe analysis was used to find high-dimensional biomarkers in jejunum microorganisms of different pig breeds. Functional abundance through eggNOG.

Supplementary Figure 3 The LEfSe analysis was used to find high-dimensional biomarkers in jejunum microorganisms of different pig breeds. Functional abundance through CAZy.
