# Supplementary figures and images for: Comparative Analyses of Antibiotic Resistance Genes in Jejunum Microbiota of Pigs in Different Areas
Source: Front Cell Infect Microbiol. 2022 May 26;12:887428. doi: 10.3389/fcimb.2022.887428 (PMC9204423; doi:10.3389/fcimb.2022.887428)

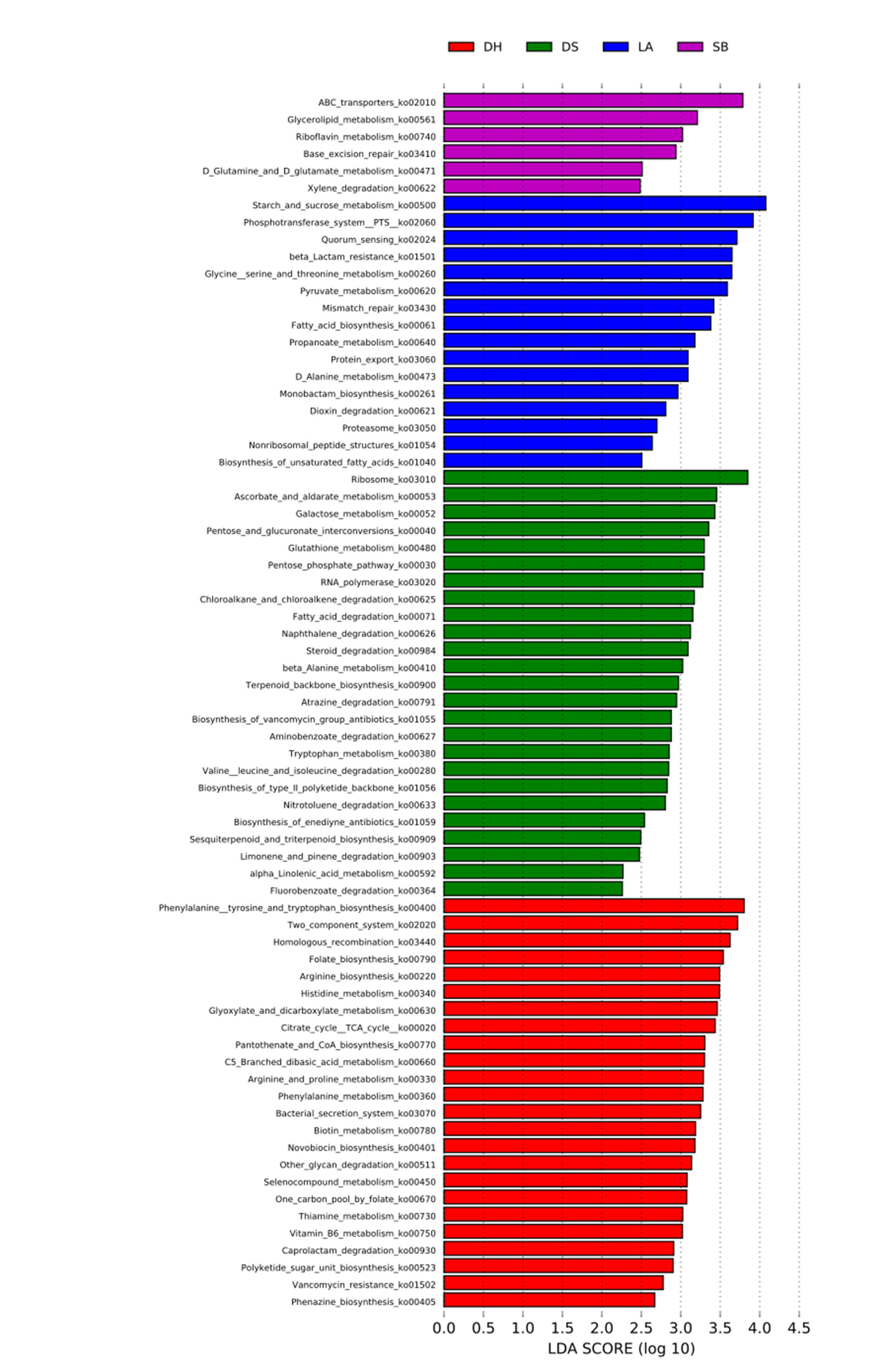

Supplement: Supplementary Figure 1 — The LEfSe analysis was used to find high-dimensional biomarkers in jejunum microorganisms of different pig breeds. Functional abundance through KEGG pathways. [file Image_1.jpeg]

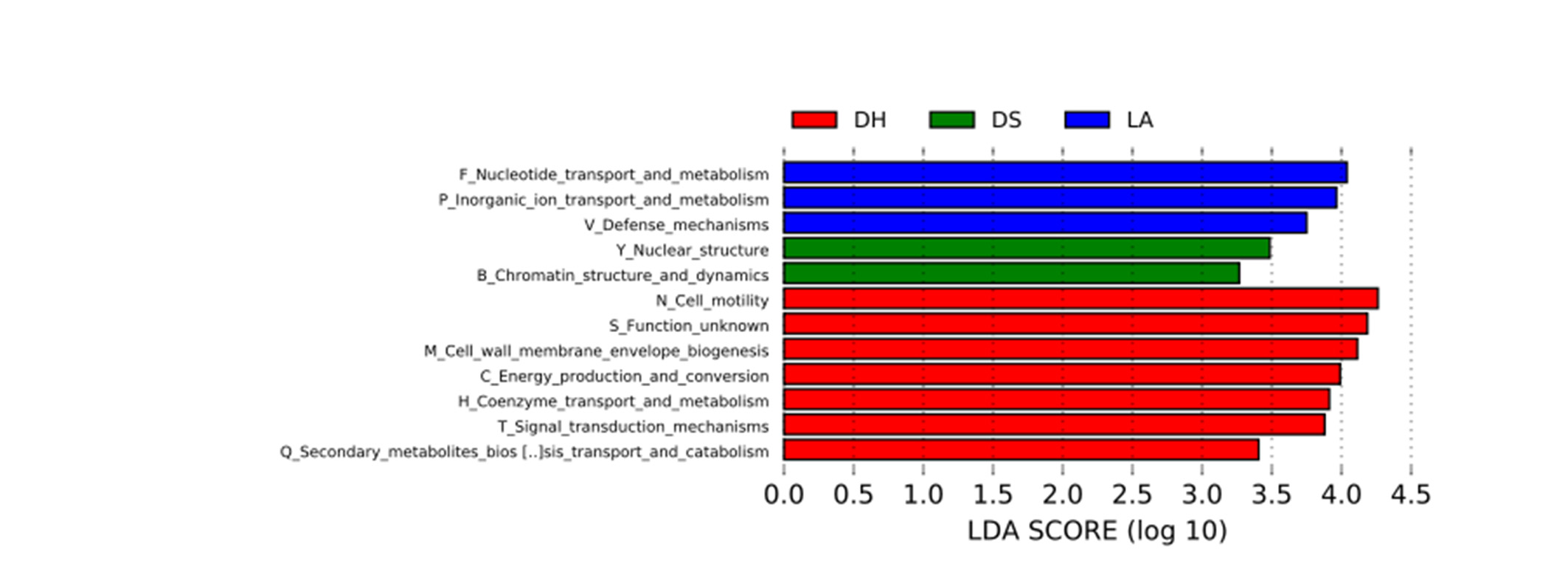

Supplement: Supplementary Figure 2 — The LEfSe analysis was used to find high-dimensional biomarkers in jejunum microorganisms of different pig breeds. Functional abundance through eggNOG. [file Image_2.jpeg]

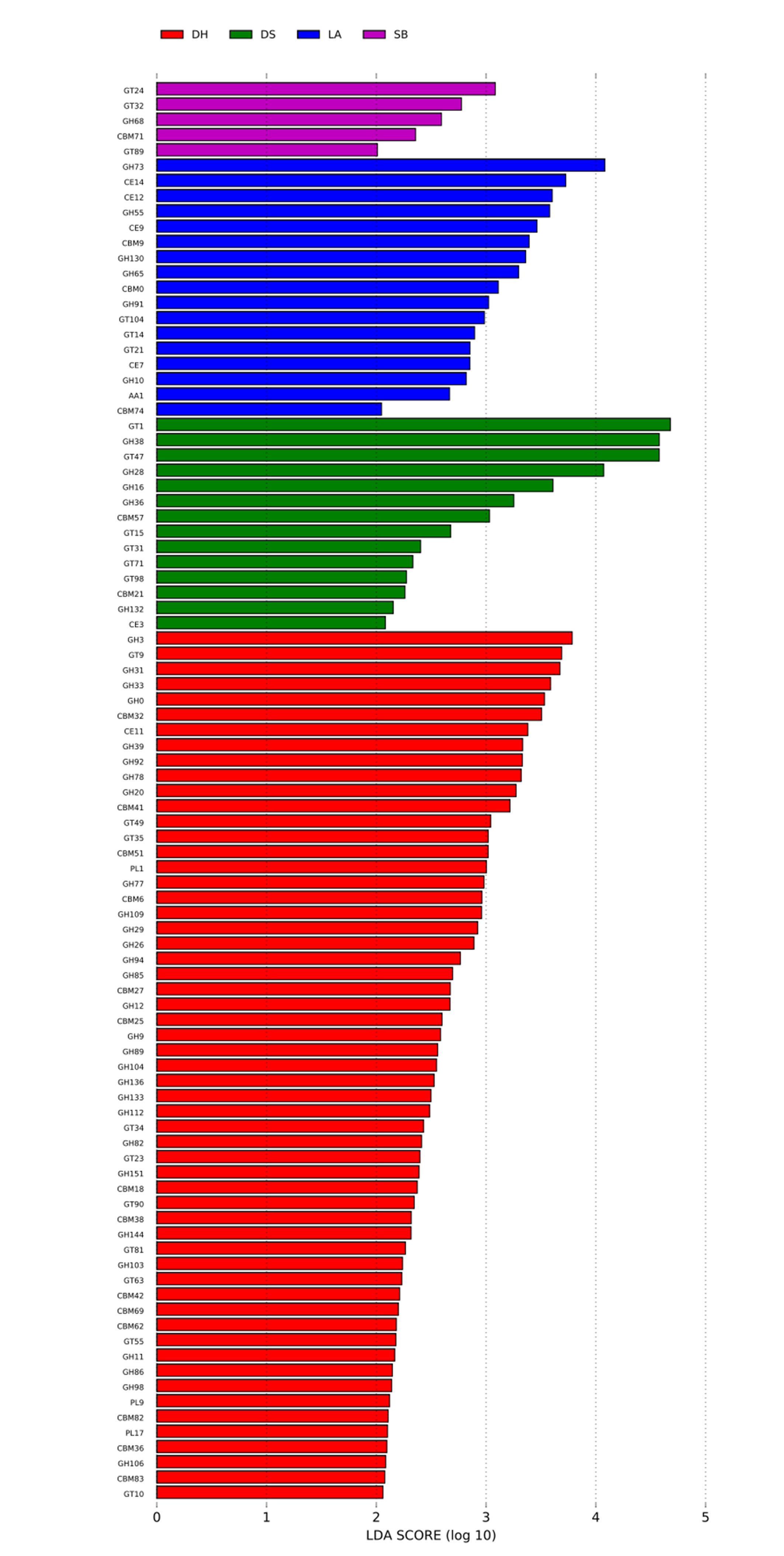

Supplement: Supplementary Figure 3 — The LEfSe analysis was used to find high-dimensional biomarkers in jejunum microorganisms of different pig breeds. Functional abundance through CAZy. [file Image_3.jpeg]
